# The effect of partner-directed emotion in social exchange decision-making

**DOI:** 10.3389/fpsyg.2013.00469

**Published:** 2013-07-25

**Authors:** Iveta Eimontaite, Antoinette Nicolle, Igor Schindler, Vinod Goel

**Affiliations:** ^1^Department of Psychology, University of HullHull, UK; ^2^Department of Psychology, York UniversityToronto, ON, Canada

**Keywords:** anger, sympathy, decision-making, social exchange, prisoner's dilemma, trust game, cognitive control, emotion

## Abstract

Despite the prevalence of studies examining economic decision-making as a purely rational phenomenon, common sense suggests that emotions affect our decision-making particularly in a social context. To explore the influence of emotions on economic decision-making, we manipulated opponent-directed emotions prior to engaging participants in two social exchange decision-making games (the Trust Game and the Prisoner's Dilemma). Participants played both games with three different (fictional) partners and their tendency to defect was measured. Prior to playing each game, participants exchanged handwritten “essays” with their partners, and subsequently exchanged evaluations of each essay. The essays and evaluations, read by the participant, were designed to induce either anger, sympathy, or a neutral emotional response toward the confederate with whom they would then play the social exchange games. Galvanic skin conductance level (SCL) showed enhanced physiological arousal during anger induction compared to both the neutral and sympathy conditions. In both social exchange games, participants were most likely to defect against their partner after anger induction and least likely to defect after sympathy induction, with the neutral condition eliciting intermediate defection rates. This pattern was found to be strongest in participants exhibiting low cognitive control (as measured by a Go/no-Go task). The findings indicate that emotions felt toward another individual alter how one chooses to interact with them, and that this influence depends both on the specific emotion induced and the cognitive control of the individual.

## Introduction

Economic theory commonly follows a normative approach to understanding human decision-making. That is, humans are assumed to be rational beings, motivated purely by the goal of maximizing gains and minimizing losses (Camerer, 1997). Recently, however, economists have taken a more descriptive approach, incorporating psychological findings of the way humans actually behave into their models. Since humans must commonly make decisions within a social context, it is important to explore the factors that influence our social decision-making. It has been found that social interactions are driven not only by logic (Camerer, 1997; Burks et al., 2003; DeSteno et al., 2010) but also by factors such as descriptive framing (Camerer, 1997; De Martino et al., 2006), fairness equilibrium (Camerer, 1997), consideration of the beliefs and desires of other players (Dubey et al., 1987; Mellers et al., 2010), perceived trustworthiness (Cox, 2004; King-Casas et al., 2005; Charness et al., 2011), and other aspects of the players' perceived character (De Dreu and McCusker, 1997). Moreover, social decision-making is influenced by our emotions (Frank, 1988; Elster, 1999). While normative economic theories rely on the view that humans are purely rational agents computing the best possible outcome, descriptive economic theories are beginning to incorporate emotion into their models (Frank, 1988; Loewenstein, 2000; Lerner et al., 2004; Andrade and Ariely, 2009).

The role of emotion in social decision-making can be explored using social exchange games. The Prisoner's Dilemma and the Trust Game are two games which are commonly used to measure decision-making in which the outcome depends on the interaction between two players. In the Prisoner's Dilemma [developed by Flood and Dresher in 1950 (Kuhn, 2009)], each of two players simultaneously choose to cooperate or to defect against the other player. If one player cooperates and one defects, then the defector wins money while the cooperator loses money. If both players choose to defect, then both will lose money, but the amount lost is less than if one is the sole defector. The pay-off matrix is such that the “rational” choice (in a one-shot game) is to defect; however in a repeated game a better outcome is received by both players when they both cooperate. In the Trust Game (Berg et al., 1995), the two players make their decisions sequentially. The first participant must choose either to cooperate and share an amount of money with the other player (in this case the amount of money the other player receives is multiplied by a certain coefficient), or to defect and keep everything for themselves. If they choose to cooperate, the other player can then either reciprocate by returning half of the received money or they can keep everything for themselves. The fact that the Prisoner's Dilemma involves simultaneous interaction, while the Trust game involves sequential choices, may result in the two games loading differently on the decision-makers' cognitive resources. Specifically, players of the Prisoners Dilemma must keep in mind four possible outcomes of the interaction and to anticipate what their opponent might chose, while players of the Trust Game must consider only two possible options and have greater influence on the end result of interaction. Cognitive load is known to influence the level of cooperation in such socially-interactive decision games. For example, when participants must memorize 7 digits (high-cognitive load) instead of 2 digits (low load), they are found to cooperate more in the Prisoner's Dilemma, particularly as the end of the game approaches (Duffy and Smith, 2012).

From studies investigating decision-making in the Prisoner's Dilemma and the Trust Game it is evident that people do not always make the “rational” choice (Dawes and Thaler, 1988). One possible factor explaining these deviations from rationality is that emotions influence our decisions in these games (Frank, 1988; Elster, 1999). As such, participants may be seen to make decisions more so with an aim of regulating their emotional responses than to maximize monetary reward. Moreover, emotions can also aid decision-making by providing information relevant for choice valuation, motivating those behaviors which are most in line with personal values as well as moral and social norms (Peters et al., 2006a; Pfister and Böhm, 2008). Emotions can also focus the decision-maker's attention onto the most salient (or personally relevant) aspects of the decision scenario, thus adjusting which information will be used most for the decision (Pfister and Böhm, 2008).

Another factor influencing what choices individuals make is cognitive control capacity. In a study by De Neys et al. (2011) performance on the Go/no-Go task was compared between individuals who rejected a high number of unfair offers in the Ultimatum Game with those who rejected a low number of unfair offers. The results showed that those who rejected a low number of unfair offers had higher cognitive control than those who rejected a high number, suggesting that judgments of fairness have a greater effect on choice behavior when cognitive control is low. Cognitive control also has influence in logical reasoning, where individuals with higher cognitive control are found to reason in line with logic while low cognitive control participants make their choices more intuitively (Stanovich and West, 2000). In addition to this, an imaging study with the Ultimatum Game showed that recipients of unfair offers had a higher activation in brain areas related to cognitive control (dorsolateral prefrontal cortex) and emotional processing (anterior insula) (Sanfey et al., 2003) suggesting that both cognitive control and emotion processing are involved in making decisions in economic games.

Here we explore the effects of two partner-directed emotions predicted to influence social exchange decision-making—sympathy and anger. Sympathy is defined as an emotional response that results from awareness of another person's undesirable experiences. Its subjective experience consists of feelings of sorrow and concern for the other, and is also associated with heightened awareness of the plights of others, and a desire to help (Eisenberg and Strayer, 1987; Eisenberg, 1991). Many researchers have considered sympathy and empathy as synonyms (Rosenberg and Towers, 1986; Eisenberg and Miller, 1987; Eisenberg and Fabes, 1990; Decety and Chaminade, 2003) and here we also do not distinguish them. On the other hand, anger is related to hostility and aggression, and varies in intensity from mild irritation to fury or rage (Spielberger et al., 1983). For sympathy to be induced, past studies have shown that the subject must adopt the other's perspective or to place at least a moderate value on the welfare of the other (Smith, 1992; Lishner et al., 2011). For anger to be induced unexpected and apparently real frustrating events, with negative impacts on wellbeing, are required (Stemmler, 1997; Clore and Centerbar, 2004; Lobbestael et al., 2008; Winterich et al., 2010; Deffenbacher, 2011).

With their differential antecedents, it is unsurprising that sympathy and anger promote differential behavioral tendencies. Sympathy has been known to induce helping behavior in students sharing their lecture notes with another student for whom illness has prevented them from taking their own notes (Reisenzein, 1986). It also promotes willingness to help a family whose son has cancer (Harmon-Jones et al., 2003) and to help a multiple sclerosis patient even after receiving an insulting comment from him (Harmon-Jones et al., 2004). Sympathetic concern also encourages higher donations when a victim (a starving child in Africa) is identifiable (where participants receive a photo and description of the child), than when the victim is presented as a non-identifiable single victim or merely as a statistic (Small et al., 2007). It is also found to encourage more generous decisions toward the other person in economic decision-making games when the outcome of interaction depends on two individuals, such as in the Prisoner's Dilemma (Batson and Moran, 1999; Batson and Ahmad, 2001; Duersch and Servatka, 2007) and “Ring Measure of Social Values” (Van Lange, 2008). In these two games, higher cooperation rates are promoted when participants perceive their opponent to be in need and when they adopt their opponent's feelings (Batson and Moran, 1999; Van Lange, 2008). In a study by Batson and Moran (1999), relating to and being aware of a partner's current difficulties, results in higher cooperation in the Prisoner's Dilemma, compared to a control condition. In a follow-up study by Batson and Ahmad (2001), this increased cooperation was apparent even when the opponent had made previous decisions in the game that were against the interests of the participant.

In contrast, anger is found to encourage higher defection rates in social-exchange games, including the Power-to-Take Game (Bosman and van Winden, 2002; Ben-Shakhar et al., 2004), the Prisoner's Dilemma (Duersch and Servatka, 2007) or the Ultimatum Game (Sanfey et al., 2003). Using the Power-to-Take Game, Bosman and van Winden (2002) found that the more anger participants felt about their opponent's decision, the more often they destroyed income even if that was costly to the participant themselves. Moreover, the intensity of felt anger is found to be positively related to the defection rate in an economic game with punishment (De Quervain et al., 2004). In addition, it has been found that anger, induced through perceptions of character, elicits violent behavior toward the anger-inducing individual (Harmon-Jones and Sigelman, 2001). Similar results emerge from studies investigating the effect of emotion on negotiation decisions. In a study by Van Kleef et al. (2004), participants acted as a phone seller and were asked to negotiate with a potential buyer about price, warranty, duration of the service contract etc., Van Kleef et al. found that, when facing angry buyers, participants made lower demands (offered lower price, longer warranty, etc.) and more often accepted bigger concessions requested by the buyer (Van Kleef et al., 2004). On the other hand, when individuals received information about the buyers' own emotional responses to either the offers or to the individuals themselves (e.g., “this [offer/person] makes me really angry”), anger directed toward their behavior was found to have different effects compared to emotions directed toward the person. Specifically, anger induced by the individual's previous offers resulted in larger concessions and lower demands compared to behavior-oriented happiness. Conversely, buyers who felt person-directed anger (i.e., buyers who said “this person makes me really angry”) encouraged individuals to make lower concessions and higher demands in the negotiation process compared to person-directed happiness (Steinel et al., 2008). In a study by Kopelman et al. (2006), participants made higher demands (when playing the role of seller), while interacting with buyers displaying negative emotions, offering higher phone price, shorter warranty period, etc., and were less likely to sign a deal compared to positive and neutral emotions (Kopelman et al., 2006). These studies not only show that anger results in reduced cooperation with others compared to other emotions (neutral and happy), but also indicate that person-directed emotions and behavior-directed emotions can have different effects on social behavior.

The current study explored how the emotions of sympathy and anger affect decision-making in the Prisoner's Dilemma and the Trust Game in a within-subject design. We hypothesized that sympathy and anger would have different effects on social decision-making, such that sympathy would reduce defection rates and anger would increase defection rates, compared to neutral emotion. Given the possibility that the two games load differently onto cognitive resources, we also explored how individual differences in cognitive control moderate emotional influences on decision-making in the Prisoner's Dilemma and the Trust Game. We expected participants with lower cognitive control to have different defection rates than those with higher cognitive control.

To test the efficacy of our emotional manipulations we used galvanic skin conductance measures and subjective reports. Skin conductance is commonly used as an indication of physiological and psychological arousal, by observing electrical conductivity responses in the skin. In accordance with past literature, we expected to find higher skin conductance levels (SCLs) to be associated with anger and sympathy emotion-induction conditions compared to neutral (Rustichini, 1966; Ben-Shakhar et al., 2004; Hein et al., 2011). We also collected self-report data and used a cluster analysis to examine the subjective experience associated with each emotion induction condition.

## Methods

### Participants

Thirty-eight participants took part in the study. All participants had normal or corrected-to-normal vision and were not undergoing any psychopharmacological treatment (one participant was removed after self-declaring that they had an anxiety disorder). Another eight participants were removed after declaring that they were aware of the deception, leaving 29 participants for the final analysis (14 females) (mean age = 23 years, *SD* = 4.4). The study was approved by the Department of Psychology ethics committee, University of Hull, and was carried out in accordance with the ethical guidelines published by the British Psychological Society, the American Psychological Association and the Declaration of Helsinki.

### Procedure

Participants were asked to come to the experiment with an essay they had written about something that was important to them. They also believed that three “other participants” had done the same and would be participating in the experiment at the same time, though the participant never met these other individuals and, indeed, they were not real. Participants were told that, for reasons of anonymity, all participants would complete the experiment in separate rooms. During the experiment participants would read the other participants' essays and would evaluate them one by one (while they believed their own essay was also being evaluated by each other participant).

Participants always began the experiment by completing the Go/no-Go task, to measure their cognitive control. Following this, they were presented with their first opponent's essay to read and evaluate. Once this essay was evaluated, participants played two distractor games while the experimenter left the room (the participant believed to collect the opponent's evaluation). The Wason Card Selection task (Wason, 1968) and the THOG task (Wason and Brooks, 1979) were used as distractors in order to make the aims of the study less obvious to participants. Performance in these tasks was not analysed further. The participant then received his opponent's evaluation of his own essay, and then immediately played three rounds of the Prisoner's Dilemma and three of the Trust Game with this same opponent. This was followed by new versions of each distractor task.

**Figure 1:**
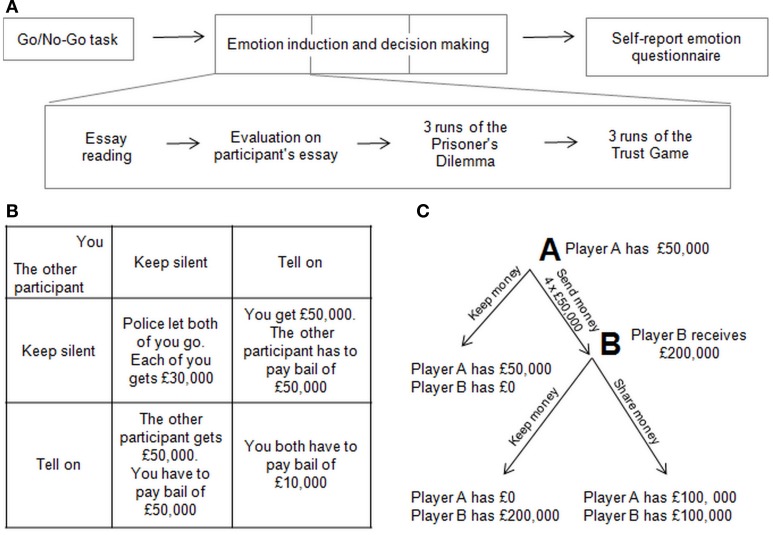
**(A)** Experiment timeline for an example participant. The order of the three emotion induction conditions, and of the two social-exchange games (the Prisoner's Dilemma and the Trust Game), were counterbalanced across participants. **(B)** Pay-off matrix for the Prisoner's Dilemma game. **(C)** Pay-off matrix for the Trust Game.

This procedure of essay reading/evaluation, distractor tasks, receipt of one's own evaluation and social-exchange game playing was then repeated for the remaining two emotion conditions (i.e., with the remaining two “other participants”). The order of emotion conditions (sympathy, anger, and neutral) and the order of the social decision-making tasks were counterbalanced between subjects to avoid order effects (Figure 1A). At the end of the experiment, participants completed the emotion questionnaire (see below). Finally, the experimenter asked questions to determine whether the participant suspected deceit or the aim of the experiment. While deception/harm to the participant was transitory, full debriefing, and contact details for a university counselor were given to participants at the end of the experiment.

### Stimuli

#### Emotional manipulation

The emotion manipulation was achieved by presenting participants with pre-constructed essays, which they believed were written by their partner participants, and with subsequent evaluations of the participant's own essays, which they believed were also written by their partners. The evaluation forms consisted of ratings of the essays on six 9-point bipolar scales (unintelligent–intelligent; thought provoking–boring; friendly–unfriendly; illogical–logical; respectable–unrespectable; irrational–rational), along with a space for free comments.

Together, the essays and evaluations were designed to induce sympathy toward one of the partner participants, anger toward another and neutral emotion toward the third. The emotion in the sympathy condition was induced through the essay, and in the anger condition was due to the negative evaluation of the participant's own essay. The sympathy-inducing essay was modified from Harmon-Jones et al.'s (2003) and concerned a young person coping with cancer. The essay was re-written according to UK education and healthcare standards. After reading this essay, the participant received a neutral evaluation of their own essay, consisting of neutral ratings (between 4 and 7 on the evaluation scales) and a hand-written positive comment “I can understand why a person would think like this.” In the anger-inducing condition the participant read a poorly written essay (grammatical mistakes, badly structured arguments) and subsequently received a negative evaluation of their own essay (Harmon-Jones and Sigelman, 2001). The anger-inducing evaluation consisted of ratings that were weighted toward negative words (e.g., illogical or unacceptable). An insulting comment was also hand-written underneath the evaluation (“This is the stupidest thing I have ever read”). In the neutral condition they received an emotionally neutral essay, written in an unemotional and grammatically correct way, followed by a neutral evaluation of their own essay (consisting of neutral evaluations between 4 and 6, and no additional hand-written comments).

The three essays/evaluations were written in clearly differentiable handwriting, and were piloted before the study to check that they triggered the targeted emotion (15 participants were monitored with galvanic skin conductance measurement and later reported what emotions the essays triggered). Galvanic skin conductance serves as an objective measure of emotional arousal, since participants cannot exert top-down control on their skin conductivity responses (Ben-Shakhar et al., 2004; Lin et al., 2005). However, we realize that a drawback of such measures is that they do not allow us to address the subjective content and direction of the emotional experience which is why we also included a self-report emotion questionnaire which participants completed at the end of the experiment.

***Self-report emotion questionnaire***. Here participants were presented with a list of 36 emotion words and, for each word, indicated which (if any) “other participant” they had felt it toward. The questionnaire was analysed with a hierarchical and k means cluster analysis.

***Galvanic skin conductance***. Galvanic skin conductance was continuously recorded through the experiment using a second computer, connected to a Biopack MP100A digital skin conductance amplifier with a constant voltage of 0.5 V. Electrodes were placed on the non-dominant hand and attached to the medial phalanx surfaces of the middle and index finger. An electrodermal gel (GEL101) was used as an electrolyte for conductance.

Galvanic SCL was calculated individually for each emotion-induction condition. The skin conductance measurements were analysed from the time when participants received the essays and evaluations (with baselines collected at rest periods before each of these critical time windows). That is for the sympathy condition, SCL was analysed while participants read the sympathetic essay and for the anger condition while reading the negative evaluation on the participant's own essay. For the neutral condition, galvanic skin conductance was averaged from reading the neutral essay and receiving the neutral evaluation on the participant's own essay. The mean SCLs were computed for each condition, using Acknowledge 3.9.1 for Windows.

#### Decision-making tasks

The following tasks were completed by participants separately for each emotion condition (with three repetitions of each task per fictional partner). The tasks were presented on a computer, using Cogent 2000v1.32 (www.vislab.ucl.ac.uk) through Matlab (version R2011.a). Participants were guided through the rules of these games, and the experimenter asked questions to make sure that the participants understood the game. To reduce participant's expectations and any reputation effects in the games, participants were told that they may or may not play some games more than once.

***Prisoner's Dilemma***. The task was developed by Flood and Dresher in 1950 (Kuhn, 2009). Participants are asked to imagine that they are two criminals who are hiding money. They have been caught by the police, separated, and each given two options: betray/defect or keep silent/cooperate. If one cooperates and the other one defects, the defector is able to keep all the money, while the cooperating player must pay a fine. If both remain silent, however, they both get half of the money. If both choose to defect, they will both have to pay half of the fine. This pay-off matrix is illustrated in Figure 1B.

***Trust Game***. In the Trust Game (Berg et al., 1995) participants can be either player A or player B. Player A has an amount of money and may decide to either send it to player B or to keep it all for himself/herself. If the money is sent to player B, the total is multiplied by four and then player B must choose to either send half back to player A or keep it all. During this experiment participants played both as player A and player B, with the order counterbalanced across the runs of the game. The pay-off matrix is given in Figure 1C.

#### Cognitive control task

Participants also completed a Go/no-Go task to measure cognitive control abilities (see De Neys et al., 2011). This task was administered once at the start of the experiment (i.e., prior to any essay reading/evaluation). At trial onset, a central fixation point was shown for 500 ms followed by a single letter for 500 ms (the target letter was either “W” or “M,” counterbalanced across participants) with an intertrial interval of 1 s. Participants were instructed to respond as fast and as accurately as possible with a keypress whenever the target letter was present. A warning message appeared if they took longer than 500 ms or the response was incorrect. In total 100 trials were presented with 80% of the trials showing the target.

### Analysis

The key-dependent measures in this study were defection rates in the Prisoner's Dilemma and the Trust Game (for each game, participants could defect a total of 0, 1, 2, or 3 times per emotion-induction condition). These dependent measures are ordinal, and Kolomogorov–Smirnov and Shapiro–Wilk tests showed that the data were not normally distributed. As a result, we used non-parametric statistical tests (as has been done previously, Brosig, 2002; Falk et al., 2005). The data were analysed with a two-way mixed non-parametric design (2 cognitive control groups × 3 emotion conditions) with defection rate as the dependent variable (Field et al., 2012). This analysis was performed separately for the Prisoner's Dilemma and the Trust Game and *post-hoc* comparisons were carried out using Bonferroni corrected Wilcoxon Signed-Rank tests (two-tailed, alpha = 0.017) to explore any differences further.

The number of errors in the Go/no-Go task was used to calculate *d*′ for each participant as a measure of cognitive control ability. Using a median split, participants were divided into two groups according to this measure; a low (*d*′ = 2.21–3.08, *N* = 14) and a high cognitive control group (*d*′ = 3.24–8.60, *N* = 15). Planned Mann–Whitney U tests were then used to analyse whether the effect of emotion on social-exchange decision-making depended on between-subject differences in cognitive control, as measured by the Go/no-Go task. Within each cognitive control group, a Wilcoxon Signed-Rank test for two related samples was used to test for within-subject differences between the effects of emotion-induction condition on defection rates (Bonferroni corrected alpha = 0.017, two-tailed).

Individual SCL scores were z-transformed for subsequent analyses with a mixed design ANOVA comparing the three emotion induction conditions (within-subject) and cognitive control (between-subject). *Post-hoc* comparisons were performed using paired *t*-tests with Bonferroni corrected alpha (two-tailed, *p* = 0.017).

## Results

### Emotional manipulation

#### Galvanic skin conductance

**Figure 2:**
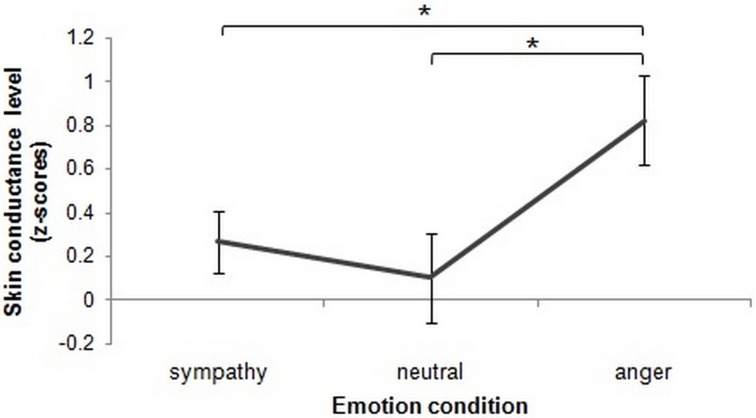
**Z-scores of mean skin conductance level (zSCL) as a function of emotion-induction.** The mean zSCL during anger-induction was significantly higher than that of the neutral induction (*p* = 0.005) and sympathy induction (*p* = 0.029). Sympathy SCL was not significantly higher than that of the neutral condition (*p* > 0.05). The asterisks highlight significant paired comparisons after Bonferroni correction (*p* ≤ 0.017). Error bars represent ±1 SEM.

A significant main effect of emotion condition on z-scored galvanic SCL (zSCL) was found [*f*_(2, 50)_ = 6.13, *p* = 0.004]. However, there was no main effect of cognitive control and there was no emotion condition × cognitive control group interaction (*p* > 0.05). *Post-hoc* analyses with paired *t*-test revealed that zSCL during the sympathy condition did not differ significantly from the neutral condition (*p* > 0.05). However, in the anger condition zSCL was significantly higher compared to the sympathy condition and to the neutral condition [*t*_(28)_ = 2.63, *p* = 0.014, and *t*_(28)_ = 4.12, *p* ≤ 0.001, respectively, Bonferroni corrected]. These findings show that anger induction, but not sympathy, is associated with a higher zSCL compared to the neutral emotional induction (Figure 2).

In order to evaluate whether zSCL was related to the effect of cognitive control on defection rate, Spearman's correlation analyses were performed separately for low and high cognitive control individuals. There were no significant correlations between defection rate and zSCL neither in high nor low cognitive control participants (*p* > 0.05).

#### Self-report questionnaire

**Figure 3:**
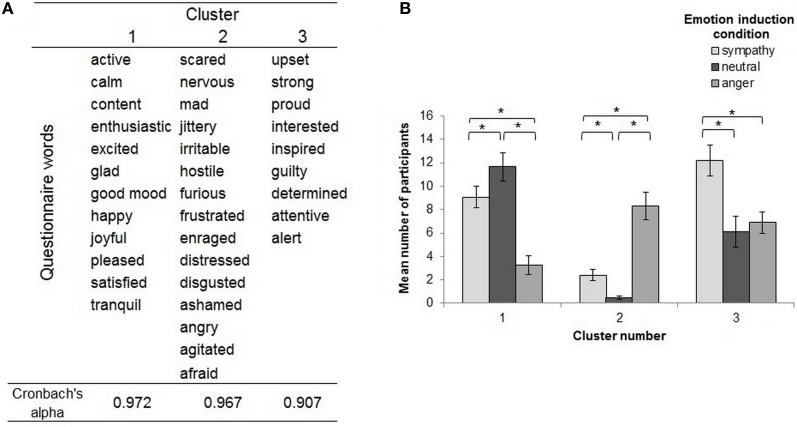
**(A)** The table shows all 36 words from the self-report emotion questionnaire grouped into three different clusters identified by the results of the cluster analysis, along with each cluster's associated Cronbach's alpha. **(B)** For each cluster of words (as identified by the cluster analysis), the figure shows the average number of participants who reported experiencing those words during the sympathy, anger, and neutral emotion conditions. The asterisks highlight significant paired comparisons after Bonferroni correction (*p* ≤ 0.017). Error bars represent ±1 SEM.

We used a hierarchical cluster analysis procedure to determine the number of clusters that could be extracted from participants' responses on the self-report emotion questionnaire. This analysis was based on the Squared-Euclidian distance following Ward's method (Willebrand et al., 2002; Bigne and Andreu, 2004) and determined the number of clusters according to an agglomeration schedule as suggested by Burns and Burns (2008). We selected a three cluster solution, on the basis that adding further clusters had minimal additional effect on the agglomeration coefficient. Accordingly, a three cluster analysis was then performed using a *k* means approach, which grouped all 36 self-report emotion questionnaire items according to their similarity across participant ratings (Bigne and Andreu, 2004). The words found to be associated with each cluster are presented in Figure 3A, along with each cluster's Cronbach's alpha. Figure 3B illustrates these clusters according to the number of participants reporting words specific to each cluster in each emotion condition. *T*-tests showed that words from cluster 1 were more often reported to be experienced during the neutral condition than the anger [*t*_(11)_ = 7.18, *p* = 0.015, Bonferroni corrected] or sympathy conditions [*t*_(11)_ = 2.89, *p* ≤ 0.001]. In contrast, words from cluster 2 were more often experienced during the anger condition, compared to the sympathy and neutral condition [*t*_(14)_ = 4.38, *p* = 0.001 vs. *t*_(14)_ = 6.94, *p* ≤ 0.001]. Cluster 3 words were more often reported in the sympathy condition than in the anger [*t*_(8)_ = 3.07, *p* = 0.015, Bonferroni corrected] or neutral condition [*t*_(8)_ = 4.06, *p* = 0.004].

### Social exchange tasks

#### The prisoner's dilemma

**Figure 4:**
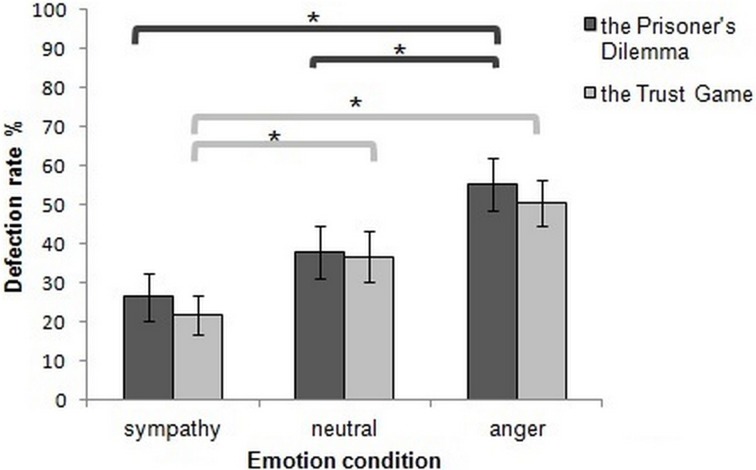
**Mean percentage defection rates in both the Prisoner's Dilemma and the Trust Game depended upon emotion condition.** The asterisks highlight significant paired comparisons after Bonferroni correction (*p* ≤ 0.017). Error bars represent ±1 SEM.

The 2 (high and low cognitive control) × 3 (anger, sympathy, and neutral conditions) mixed design non-parametric analysis yielded a significant main effect of emotion (*Q* = 0.454, *p* = 0.002) and a significant interaction between cognitive control and emotion (*q* = 5.06, *p* = 0.01). The main effect of cognitive control was not significant (*p* > 0.05). *Post-hoc* Wilcoxon Signed-Rank tests showed a significantly higher defection rate after anger induction compared to sympathy induction (*Z* = −3.21, *p* = 0.001, Bonferroni corrected). While there was no significant difference between the defection rates following neutral and sympathy induction (*p* > 0.05), the anger induction resulted in higher defection rates compared to the neutral induction (*Z* = −2.84, *p* = 0.004, Bonferroni corrected) (Figure 4).

**Figure 5:**
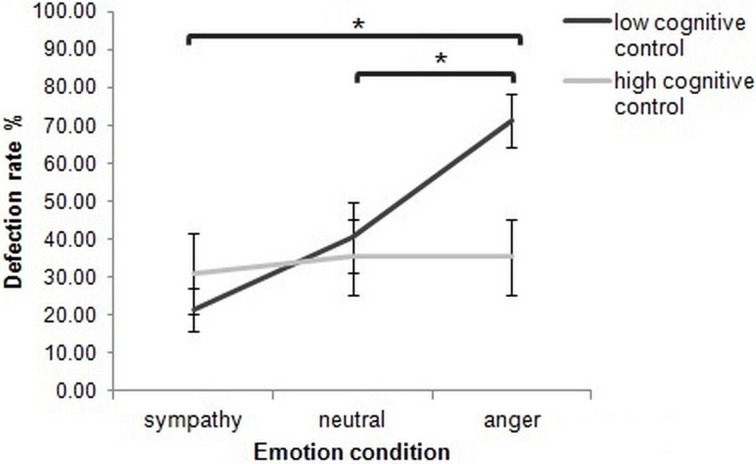
**In the Prisoner's Dilemma, the effect of emotion on mean percentage defection rate depended upon cognitive control group.** The asterisks highlight significant paired comparisons after Bonferroni correction (*p* ≤ 0.017). Error bars represent ±1 SEM.

To explore the interaction effect further, within-subject comparisons with Wilcoxon Signed-Rank tests were then performed for each cognitive control group separately. Defection rates did not differ significantly between emotion-induction conditions in high cognitive control participants (*p* > 0.05). In contrast, low cognitive control participants showed a significantly higher defection rate in the anger condition, compared to both neutral and sympathy inductions (*Z* = −2.98, *p* = 0.003, and *Z* = −2.90, *p* = 0.005, respectively, Bonferroni corrected). The increased defection rate for the neutral, compared to the sympathy condition, was not significant (*p* > 0.05) (Figure 5).

#### The trust game

The same non-parametric mixed design analysis was performed for the Trust Game. The results showed a significant main effect of emotion (*Q* = 9.10, *p* = 0.001), but no main effect of cognitive control and no significant cognitive control × emotion interaction (*p* > 0.05). *Post-hoc* comparisons with Wilcoxon Signed-Rank tests yielded a significantly higher defection rate in the neutral condition compared to sympathy induction (*Z* = −2.45, *p* = 0.014, Bonferroni corrected) and a significantly higher defection rate after anger induction compared to sympathy induction (*Z* = −3.36, *p* = 0.001, Bonferroni corrected). The difference in defection rates between the neutral and anger conditions was not significant (*p* > 0.05) (Figure 4).

#### Additional analyses

Kruskall–Wallis tests for more than 2 independent samples did not find any influence of emotion-induction order on participants' defection rates for either game (*p* > 0.05). Additionally, defection rates did not differ depending on the “other participants” previous choice (defect or cooperate) in either game for any emotion (Wilcoxon Signed Ranks; *p* > 0.05).

The asymmetry of the effects of anger and sympathy (compared to neutral) on defection rates were tested using Wilcoxon Signed-Rank tests. There was no significant interaction effect of [anger – neutral] vs. [neutral – sympathy] for either game (*p* > 0.05). These results suggest that, despite sympathy and anger exerting opposite effects on decision-making (compared to neutral), the relative strength of these effects was symmetrical.

Finally, to evaluate whether the Prisoner's Dilemma and the Trust Game have different cognitive demands, the overall defection rates in both games were compared. Although participants chose defection more often in the Prisoner's Dilemma than in the Trust Game, the Wilcoxon Signed Ranks test did not reveal a significant difference between the cognitive control groups; neither in overall defection rates nor separately in each emotion condition (*p* > 0.05).

## Discussion

This study investigated the influence of partner-directed emotions on social decision-making. The experiment compared the effects of two emotion inductions (anger and sympathy) and one baseline (neutral) emotional condition, and assessed their differential impacts on decision-making in two social-exchange games—the Prisoner's Dilemma and the Trust Game.

The results of the self-report questionnaire indicated that the three emotion induction conditions were associated with distinct affective experiences. The feelings most associated with the anger induction were all negative and in keeping with common definitions of anger (see Figure 3). The cluster most associated with our sympathy induction included a mix of positive and negative feelings, suggesting that sympathy may be a more complex (or mixed) emotional experience. Specifically, some of the feelings are associated with empathic understanding of others (e.g., upset and also feeling strength in the knowledge that people can cope with a disease), while others may be more linked to heightened concern for others (e.g., feeling attentive and alert), or with the effect the other person's psychological state has on oneself (e.g., feeling inspired and interested). The cluster of feelings most strongly associated with the neutral condition was positive and relatively placid, which was also in keeping with our expectations. While this cluster was significantly more associated with the neutral condition than both emotional conditions, the sympathy condition did also load somewhat onto this cluster (clearly more so than the anger condition), suggesting that there may be a certain level of overlap between our neutral and sympathy conditions. It is worth noting, however, that the neutral condition showed no closer relationship than the anger condition with the cluster that was most associated with sympathy (i.e., Cluster 3).

Our skin conductance findings show clearer evidence of overlap between the sympathy and neutral conditions, in that our anger induction was associated with increased SCL, while our sympathy induction was not. This result is consistent with findings by Frodi and Lamb (1980) (see also Frodi et al., 2006), who showed that sympathy-oriented emotions had no significant impact on physiological responses. On the other hand, threat related stimuli such as angry faces, spiders, or snakes are detected faster due to evolutionary reasons (Öhman and Mineka, 2001; Öhman et al., 2001). This idea has received criticism, however, by those who suggest that the speeded responses to fearful or threatening stimuli are due to the relevance of the stimuli to the individual rather than its negative valence (Sander et al., 2003; Brosch et al., 2007, 2008, 2010). In the context of this study, it is possible that participants perceived the anger induction-condition to be more relevant to their current situation which resulted in a stronger emotional response and inducing a desire in participants to do something to change their feelings. In contrast, induced sympathy may not always promote such strong action tendencies. Accordingly, anger results in higher arousal, while sympathy is more neutral in terms of the evoked physiological response. Another explanation may be that sympathy *does* have a physiological impact, but that this was simply not measurable through SCL in our experiment.

The results of the social-exchange games indicated that, although the sympathy and neutral conditions did not differ noticeably in their effects on physiological arousal, both the anger and the sympathy inductions had significant (and opposing) effects on participants' social decision-making. The direction of this effect was consistent with past findings—sympathy triggered lower defection rates and anger triggered higher defection rates compared to the neutral condition (Batson and Moran, 1999; Bosman and van Winden, 2002; Ben-Shakhar et al., 2004; Duersch and Servatka, 2007; Van Lange, 2008). Moreover, the strengths of these impacts were found to be more or less symmetrical compared to the neutral condition, despite only the anger condition having significant influences on participant's physiological arousal.

Though the defection rate tended to show at least some increase from sympathy to neutral and from neutral to anger in both games, there were subtle differences between the two games: in the Prisoner's Dilemma significant differences were found between anger and neutral, and in the Trust Game between sympathy and neutral. Therefore, both games were affected by the emotion manipulations, but in slightly different ways. One possible explanation for this pattern of results could be the different framing of the choices in the games. The Prisoner's Dilemma holds a loss frame, because one possible outcome of the game is that the participant might lose money. In contrast, the Trust Game holds a gain frame, since the participant can either gain money or else they will neither lose nor gain. Framing effects have yielded conflicting results in different studies. Though there are a wide range of experiments showing that such framing does influence individuals' decisions (De Dreu and McCusker, 1997; Tversky and Kahneman, 1981; Frank and Claus, 2006) other studies find that not all people are affected by the framing effect (Peters et al., 2006b). The results of the current study hint that framing effects may interact with emotion in social decision games. In the Prisoner's Dilemma participants are generally more driven to avoid loss, and the anger condition may make these losses more salient and the option to defect even more tempting. Conversely, the Trust Game rewards cooperation, and this may be further promoted by sympathy rather than anger. Future studies could explore these possible effects of framing on the influence of emotion in social decision-making.

A particularly interesting finding from this study is that the effect of anger on decision-making in the Prisoner's Dilemma depended on cognitive control ability—as determined by performance in a Go/no-Go task. The effect of anger was driven almost exclusively by the low cognitive control group. This is consistent with De Neys et al. (2011), who found that participants showing high defection rates in the ultimatum game also made more mistakes in a Go/no-Go task, compared to the low defection rate participants. Our SCL analysis, however, did not indicate a difference in the strength of experienced emotions between low and high cognitive control participants. It is possible that high cognitive control participants were better at focusing on the game itself, and were therefore less affected by their emotions. Kollock (1998) as well as Komorita and Parks (1999) note that, in the long-term, cooperation can bring bigger benefits to the players than defection, and high cognitive control participants may be more likely to use this logic while playing the game. On the other hand, low cognitive control participants may be relying more on intuition (Stanovich and West, 2000; Sunstein, 2005), and in particular an “outrage heuristic,” which promotes a desire to punish others as retribution for their anger (Kahneman and Frederick, 2002).

One strength of the present study lies in its within-subject design, whereby the influence of both emotions—sympathy and anger—were measured and compared to a neutral baseline within the same group of participants. The value of a within-subject design results particularly in the reduction in variance when comparing our emotional manipulations. In between-subject designs, such comparisons may be confounded by variance due to individual differences or context effects, giving us less power to address the effect of the emotional responses we are interested in. Moreover, in exploring the effect of our between-subject measure of cognitive control, a within-subject emotional manipulation allows us to address not only the role of cognitive control on the effect of one emotion (e.g., anger) on decision-making, but importantly to address its role in the *change* in decision-making between two emotion conditions. In addition to this, the study assessed the influence of emotions directed *toward* the other player with whom participants were playing, rather than being purely incidental to the decision-scenario. To our knowledge, this is the first study that uses a within-subject design for investigating two different emotions directed toward the other person.

One limitation to the current study may be possible reputation effects induced through multiple repetitions of the games. Although game order was counterbalanced, and we did not find effects of reputation in the three sequential runs of each game, future studies might randomize the trials completely, such that participants play multiple games against the three partners in a fully interleaved manner. Another limitation may be in the self-report emotion questionnaire used, where sympathy and anger conditions had different amounts of words representing the possible emotions (indeed while the word “anger” was present in the list, “sympathy” was not). Finally, our data show that anger and sympathy differ in their experiential complexity and associated physiological arousal, as well as in their valence. As such, it is yet unclear precisely which of these components of anger and sympathy best explain their differential effects on social decision-making. Future studies could directly compare the motivational effects of emotions that are of similar levels of experiential complexity but differ in terms of valence and/or arousal, or conversely that are of similar valence and arousal but differ in their complexity.

This study shows the differential effects of sympathy and anger (directed toward the opponent) on socially-interactive decision-making. Emotions can be beneficial when making decisions—especially when people do not have time to consider all the possible choice options and their possible outcomes carefully. Specifically, emotions can help us to solve a problem more efficiently, and in better accordance with our personal goals and moral and social norms, than can decision-making in the absence of emotional influence (Peters et al., 2006a; Pfister and Böhm, 2008). Indeed, the results of this study show that sympathy and anger, directed toward ones opponent, can have emotion-specific influences on our social interaction, further reflecting the goal-directed nature of emotion influences on decision-making. If a person feels angry, and is motivated to use this emotion in the decision process, their tendency to defect increases. In contrast, if they are motivated to help their partner (as is typical of sympathy) then their level of co-operation will increase. In our Prisoner's Dilemma game, healthy individuals with higher cognitive control tended to rely less on their anger felt toward others in their decision-making, while individuals with lower cognitive control tended to be more heavily influenced by feelings of anger and chose to defect more often, perhaps as punishment or to express their anger. These findings provide support for complex, and likely bidirectional, interactions between emotion and cognition in decision-making. Heuristic-based thinking styles have also been suggested to account for judgments and decisions made in many moral and social contexts (Sunstein, 2005) such as in the Trolley Dilemma, emission trading or Asian Disease problem. Moreover, emotion-based heuristics (or “affect heuristics”) have been proposed to provoke judgments and decisions that are heavily biased by our emotional responses without the involvement of significant cognitive deliberation (Slovic et al., 2007). In accordance with such accounts, feelings of anger would be expected to provoke behaviors that can express this anger and seek retribution (as can be done through defecting). On the other hand, sympathy promotes a desire to help the person in need, and this motivation leads to enhanced co-operation. In keeping with accounts that emotions can bias judgment and decision-making through a heuristic route, our findings suggest that people who are more likely to utilize heuristic processing styles (as in the case of our low cognitive control participants) will be more heavily influenced by their emotional responses.

### Conflict of interest statement

The authors declare that the research was conducted in the absence of any commercial or financial relationships that could be construed as a potential conflict of interest.
